# The effects of freeze-dried *Ganoderma lucidum* mycelia on a recurrent oral ulceration rat model

**DOI:** 10.1186/s12906-017-2021-8

**Published:** 2017-12-01

**Authors:** Ling Xie, Xiaohong Zhong, Dongbo Liu, Lin Liu, Zhilan Xia

**Affiliations:** 1 College of Horticulture and Landscape Architecture, Hunan Agricultural University, Changsha, China; 2 State Key Laboratory of Subhealth Intervention Technology, Changsha, China; 3 The First Affiliated Hospital of Hunan University of Chinese Medicine, Changsha, China; 4 Hunan Engineering Research Center of Edible Fungi, Changsha, China

**Keywords:** Freeze-dried powder from Ganoderma lucidum mycelia, Rrecurrent oral ulcer, Rregulatory T cells, T-helper cells 17

## Abstract

**Background:**

Conventional scientific studies had supported the use of polysaccharides and β-glucans from a number of fungi, including Ganoderma lucidum for the treatment of recurrent oral ulceration (ROU). Our aim of the present study was to evaluate whether freeze-dried powder from *G. lucidum* mycelia (FDPGLM) prevents ROU in rats.

**Methods:**

A Sprague-Dawley (SD) rat model with ROU was established by autoantigen injection. The ROU rats were treated with three different dosages of FDPGLM and prednisone acetate (PA), and their effects were evaluated according to the clinical therapeutic evaluation indices of ROU.

**Results:**

High-dose FDPGLM induced significantly prolonged total intervals and a reduction in the number of ulcers and ulcer areas, thereby indicating that the treatment was effective in preventing ROU. Enzyme-linked immunosorbent assay (ELISA) showed that high-dose FDPGLM significantly enhanced the serum transforming growth factor-β1 (TGF-β1) levels, whereas reduced those of interleukin-6 (IL-6) and interleukin-17 (IL-17). Flow cytometry (FCM) showed that the proportion of CD4^+^ CD25^+^ Foxp3^+^ (forkhead box P3) regulatory T cells (Tregs) significantly increased by 1.5-fold in the high-dose FDPGLM group compared to that in the rat model group (*P* < 0.01). The application of middle- and high-dose FDPGLM also resulted in the upregulation of Foxp3 and downregulation of retinoid-related orphan receptor gamma t(RORγt) mRNA.

**Conclusion:**

High-dose FDPGLM possibly plays a role in ROU by promoting CD4^+^ CD25^+^ Foxp3^+^ Treg and inhibiting T helper cell 17 differentiation. This study also shows that FDPGLM may be potentially used as a complementary and alternative medicine treatment scheme for ROU.

**Electronic supplementary material:**

The online version of this article (10.1186/s12906-017-2021-8) contains supplementary material, which is available to authorized users.

## Backgroud

Recurrent oral ulceration (ROU), which is one of the most common complications of acquired immune deficiency syndrome (AIDS), Behcet’s disease, and malignant tumors, is the most prevalent oral mucosal disease occurring around the world. Although its etiology and pathogenesis remain unclear, ROU meets most characteristics of autoimmune diseases (ADs) such as immune disorders, repeated attacks, and self-limitations. Furthermore, certain autoantibodies, including antinuclear antibodies (ANAs) and circulating immune complexes (CICs,) could be detected in ROU [1]; therefore, ROU is generally classified as an AD. No ideal drugs and treatment schemes to control ROU have been established to date. Despite the availability of several treatment approaches by conventional and complementary western medicine, their efficacy is often unsatisfactory. Hence, Traditional Chinese Medicine (TCM) or medicinal herbs have frequently been used for the treatment of ROU [2].

*Ganoderma lucidum*, a popular medicinal fungus commonly known as “Lingzhi” in China and “Reishi” in Japan, has been historically employed as a TCM and complementary medicine. The bioactive substance in *G. lucidum* is generally isolated from the fruiting body of artificial cultures,mainly including some water-soluble polysaccharides [3]. Accoring to Yihuai Gao study, the refined fraction above consisted of glucose (61.2%), xylose(15.5%), galactose (4.8%), fructose (14.4%) and rhamnose(4.1%) linked together by β-glycosidic linkages [4]. The increase in the demand for the bioactive substance of *G. lucidum* has resulted in the need to shorten the five-month cultivation period of this fungal species. Liquid cultures of *G. lucidum* mycelia may significantly shorten its culture time and facilitate in the isolation of the bioactive substances [5]. Modern pharmaceutical and nutritional research studies have shown that the fruiting body and mycelium of *G. lucidum* have several physiological and therapeutic effects, including immunomodulating activities [6], enhancing immune functions [7], and antitumor activities [8]. Previous studies have shown that the bioactive substance in *G. lucidum* may be utilized in the treatment of ADs such as Crohn’s disease (CD), inflammatory bowel disease (IBD), and colitis [7, 9, 10].The polysaccharide fractions from *G. lucidum* or other fungi have been reported to have anti-ulcer effects against experimental ulcers in the rat especially gastric mucosal lesions [4, 11]. A report showed that a number of fungi, including *G. lucidum* produces β-[1, 3]-glucans had a regulatory function on lymphocyte production and function in patients with ROU [12].

The present study aimed to investigate the effect of FDPGLM on a Sprague-Dawley (SD) rat model for ROU, analyze the influence of FDPGLM on the differentiation of CD4^+^CD25^+^Foxp3^+^Tregs, and assess the expression of Foxp3 and RORγt transcription factor genes and serum level of cytokines that are associated with Th17 cells and Tregs to elucidate the mechanism of FDPGLM in ROU and provide experimental data for further investigation.

## Methods

### FDPGLM preparation

Mycelia were obtained after a laboratory solid strain G6 of *G. lucidum* (from Hunan Engineering Research Center of Edible Fungi in Changsha,identified as *G. lucidum (Leyss. ex Fr.)Karst.* via Professor Zhilan Xia) activated with potato dextrose broth (PDB), ground under aseptic conditions, inoculated quantitatively, and cultured at 28 °C with constant shaking at 180 rpm for 4 days. After culturing at 100 rpm for another 6 days, the mycelia were collected, filtered, cleaned 3–4 times by water, crushed, freeze-dried in a vacuum freeze-drier (Thermo Savant, USA), and stored at 4 °C [13, 14]. Before intragastric administration, FDPGLM dissolved in water and then ground by tissue grinder into homogenate (Additional file 1: Figure S1).

The analysis of the content of total polysaccharides and triterpenes about 5 samples by UV-VIS spectrophotometry according to Chinese Pharmacopoeia(Edition 2015). The detection wavelength was 625 nm and 546 nm respectively.Results (Additional files 2 and 3: Table S1 and S2)showed that the content of total polysaccharides was 8.40%(RSD < 5%) and 833.3% higher than the standard in Chinese Pharmacopoeia(≧ 0.9%).The content of triterpenes was 0.23%(RSD < 5%) and lower than the standard of CP(≧ 0.5%). Meanwhile, HPLC results (Additional file 4: Table S3)showed that the content of Ganoderic Acid A was 1.04‰, RSD < 5%.

### Preparation of autoantigens

SD rats (SPF grade, average weight: 200 g) were purchased from Hunan Slack King of experimental animals (Changsha, China). Rats were anaesthetized by pentobarbital through intraperitoneal injectionn before euthanization by cervical dislocation.After the SD rats sacrificed, their oral mucosal tissues were immediately stripped under aseptic conditions and slices into pieces. The mucosal tissues were homogenized in 0.1 mol/L phosphate buffered saline (pH 7.4), aliquoted, and stored at −80 °C until analysis.

### Establishment of an ROU animal model

This study was conducted in strict accordance with guidelines established by the Committee on the Use and Care of Animals at the Hunan Province, P. R. China. The experiments were approved by the Ethics Committee of the First Affiliated Hospital of Hunan University of Chinese Medicine. SD rats were housed in a room at a constant temperature (22 ± 1 °C) with 12-h light/dark cycles and fed with standard pellet chow and water ad libitum. After thawing, the homogenate was mixed with Freund’s complete adjuvant (CFA, Sigma, F5881) under aseptic conditions (1:1). One day before administration, the backs of the rats were depilated with 8% sodium sulfide (2 cm^2^ of the left and right regions of the spine). The rat ROU model was established by intradermal injection of 0.2 mL of an antigen emulsifier into the rat left and right region of the spine once a week for a total of 8 weeks, and only CFA was injected in the controls [15]. The animals were euthanized by applying three times the dosage of pentobarbital through intraperitoneal injection.

### Animal grouping and drug administration

Rat models were randomly divided into five groups. FDPGLM (100 mg/kg, 200 mg/kg, and 300 mg/kg) and PA (125 mg/kg) was administered via gavage once a day for a total of 20 days. Water was given instead of drugs in the model control group.

### Establishment of the therapeutic evaluation indices

The evaluation indices, which were established in accordance with the procedures outlined in the ROU Therapeutic Evaluation Indices Standard from the Chinese Stomatological Association (GSA) (reapproved in 2001), included the total ulcer interval (I, d), total number of ulcers (N), and size of ulcer area (mm^2^). The evaluation indices were as follows: I1: prolongation in the total interval (*P* < 0.05), I0: no change in the total interval (*P* > 0.05), N1: reduction in the total number of ulcers (*P* < 0.05), and N0: no change in the total number of ulcers (*P* > 0.05).

### Histopathological examination

Six rats from different groups were euthanized by pentobarbital and oral mucosa.was collected.Mucosal specimens were fixed in 4% paraformaldehyde for 3 days, embedded in paraffin, serially sectioned, and stained with hematoxylin-eosin. The inflammatory cells, necrosis, and ulcers were assessed under a microscope.

### ELISA

Serum cytokine levels associated with Th17 cells and Tregs (TGF-β1, IL-17, IL-10, and IL-6) were quantified by using ELISA kits that were specific for rats according to the manufacturers’ instructions (all ELISA kits from ImmunoWay Biotechnology Company, USA). The cytokine contents were expressed as pg/L or ng/L.

### FCM analysis

Peripheral blood mononuclear cells (PBMCs) were prepared by using a peripheral blood lymphocyte separation kit for rats (TBD Science, LTS1083, 200 mL/kit). To detect Tregs (cell density: 1 × 10^6^ cells) were stained with anti-rat CD4 FITC OX35 (Ebioscience, Cat. No. 11–0040-81) and anti-Rat CD25 PE (Ebioscience, Cat. No. 12–0390-80), and incubated for 30 min at 4 °C in the dark according to the manufacturer’s protocol. After rupturing the membrane by using a Foxp3 staining buffer set (Ebioscience, Cat. No. 00–5523-00), the cells were then incubated with anti-mouse/rat Foxp3 PE-Cy7 (Bioscience, Cat. No. 25–5773-80). Rat IgG2a K isotype controls PE-cyanine (Ebioscience, Cat. No. 25–4321-81) were used to compensate and confirm antibody specificity [16, 17]. The percentage of CD4^+^CD25^+^Foxp3^+^Tregs and CD4^+^T cells were counted by using a FACS Canto II (BD, USA), and the CD4^+^CD25^+^Foxp3^+^Tregs/CD4^+^T ratio was calculated.

### Real-time RT-PCR analysis

After animals were euthanized, total RNA from oral mucosa was extracted by using a RNA prep pure tissue kit (Tiangen Biotech Co., Ltd. centrifugal cylinder, China) according to the manufacturer’s instructions.The quality and quantity of the RNA were determined by ultraviolet spectrophotometry. All samples were treated with DNase to eliminate potential genomic DNA contamination. The first strand of the complementary DNA (cDNA) was synthesized using a M-MLV RTase cDNA synthesis kit (Invitrogen, USA). cDNA was then amplified using primers specific for the rat Foxp3, RORγt, and GAPDH genes. The following primer sequences were used for PCR analysis: (ROR) γt, F: 5′ GCCTACAATGCCAACAAC3’ and R: 5’TCGAATATGGAGCTGATGAG3’; Foxp3, F: 5′ ATGTTCGCCTACTTCAGAA3’ and R: 5’TCATCTACGGTCCACACT3’; and GAPDH, F: 5′ TTCAACGGCACAGTCAAG3’ and R: 5’TACTCAGCACCAGCATCA3’. The RT-PCR reactions were performed in a 25-μL volume using Platinum® SYBR® Green qPCR Super Mix-UDG(Invitrogen, USA) in accordance with the manufacturer’s protocols. All samples were analyzed using an Applied Biosystems ABI 7500 Real-time PCR (USA) system. PCR conditions for gene amplification were as follows: 2 min at 50 °C, followed by 40 cycles of 95 °C for 2 min, 95 °C for 15 s, and 60 °C for 30 s) [18]. The PCR products were analyzed by using a basic relative quantification method, and the amplification products were confirmed based on the observation of a single peak in the melting curve.

### Statistical analysis

Statistical analysis was performed using PASW 18.0 Windows and Origin 7.5 software. Values were expressed as the mean ± SD for the indicated number of independent experiments. Normality analysis was performed by using the one-sample Kolmogorov-Smirnov test, and statistical significance was determined by one-way ANOVA. *P* values < 0.05 were considered statistically significant, and *P* values < 0.01 were treated as statistically different.

## Results

### Effect of FDPGLM on the total interval, number and size of ulcers in an ROU rat model

LSD testing(Least Significant Difference testing) indicated significant differences in the total interval duration between the ROU rat model PA (*P* < 0.01) and high-dose (*P* < 0.01) groups, whereas no statistical significance (*P* > 0.05) was observed between the low- and middle-dose groups (Table 1), thereby indicating that PA and high-dose FDPGLM could prolong the ulcer interval duration (I1). Furthermore, significant differences in the number of ulcers were observed between the model and the PA groups, as well as that in the middle- and high- dose groups (*P* < 0.01).

**Table 1:** Effect of FDPGLM on the total interval, number of ulcers, and area in a ROU rat model ($\overline{x}\pm s$, *n* = 8)

Group	Interval (d)	Number	Area (mm^2^)	Evaluation
Model	51.75 ± 1.03^c3^	3.13 ± 0.45^c3^	4.50 ± 0.32^c3^	/
PA	54.03 ± 1.60^b3^	1.51 ± 0.21^b3^	3.11 ± 0.37^b3^	I1N1
Low FDPGLM	52.50 ± 1.61^b1 c1^	2.25 ± 0.17^b1 c1^	4.48 ± 0.29^b1 c3^	I0N0
Mid FDPGLM	53.88 ± 1.13^b1 c1^	1.59 ± 0.25^b3 c1^	3.75 ± 0.21^b1 c1^	I0N1
High FDPGLM	53.05 ± 1.55^b3 c1^	1.25 ± 0.19^b3 c1^	3.14 ± 0.26^b3 c1^	I1N1

① b: each group compared to the model group; c: each group compared to the PA group

② b1: *P* > 0.05, b2: *P* < 0.05, b3: *P* < 0.01; c1: *P* > 0.05, c2: *P* < 0.05, c3: *P* < 0.01

③ Statistical symbols and their meanings in following figures (Figs. 3, 4 and 5) are the same as this one

### Histological analysis of the oral mucosa

**Fig. 1:**
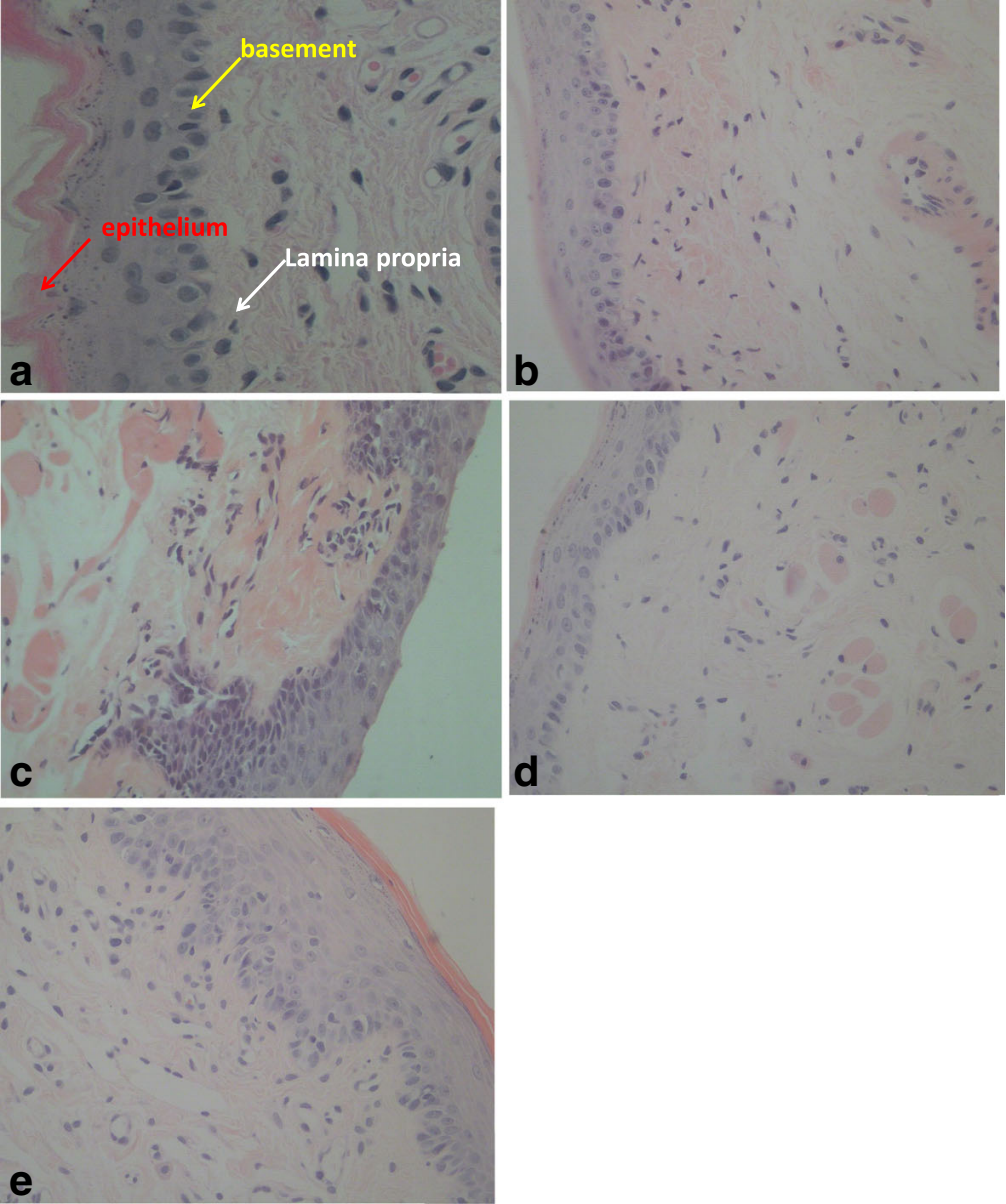
Histological changes in the oral mucosa of ROU rats (HE, 40× magnification).**a**, model group; **b**, PA group; **c**, low-dose FDPGLM group; **d**, middle-dose FDPGLM group; **e**, high-dose FDPGLM group

Figure 1A shows partial destruction of the epithelial tissue of the oral mucosa of the rat model, wherein the epithelial layer was thinner, the cells of the basement layer and the lamina propria showed irregular arrangement, and an increase in the number of inflammatory and fibroblast-like cells. After treatment of the ROU rats with the corresponding drug (PA in Fig. 1b; high-dose FDPGLM in Fig. 1e), repair of the membrane of the epithelial mucosa was observed, and the cells of the basement layer and the lamina propria exhibited normal organization with the rare inflammatory cells surrounding the blood vessels.

### The levels of cytokines associated with Th17 cells and Tregs

**Fig. 2:**
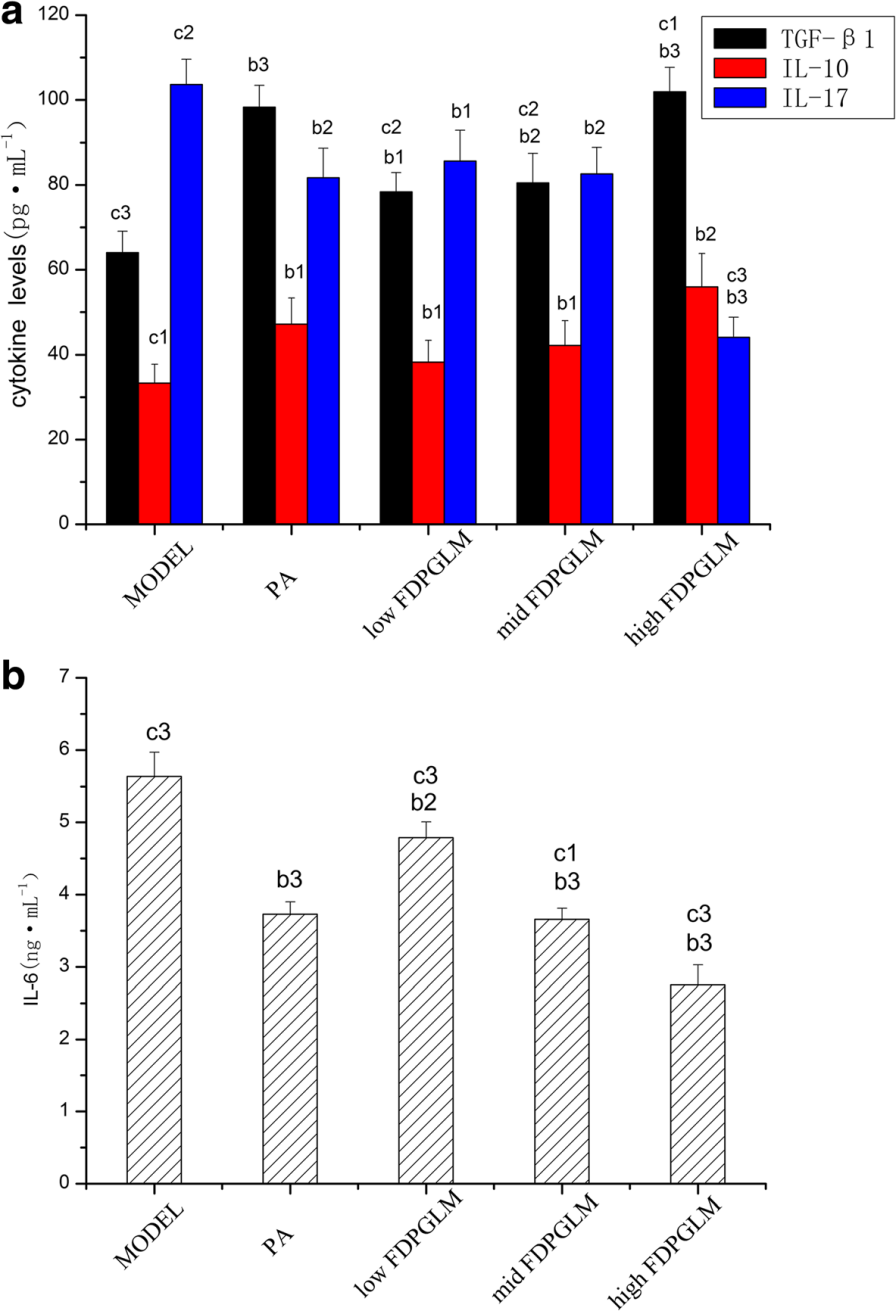
Effect of FDPGLM on serum levels of CK associated with th17 cells and Tregs. ①b, each group compared to the model group; c, each group compared to the positive drug group. ②b1: *P* > 0.05, b2: *P* < 0.05, b3: *P* < 0.01; c1: P > 0.05, c2: *P* < 0.05, c3: *P* < 0.01. ③Statistical symbols and their meanings in following figures or tables are the same as this one

We determined the levels of cytokines that were associated with Th17 cells and Tregs (TGF-β1, IL-17, IL-10, and IL-6)in peripheral blood. ELISA indicated that the levels of TGF-β1, which is a key factor that promotes wound healing [19], significantly increased (*P* < 0.01) in the PA and high-dose FDPGLM groups in contrast to the model group (Fig. 2a and b). The difference between PA group and high-dose FDPGLM group (*P* > 0.05) was not statistically significant, indicating that high-dose FDPGLM can improve level of TGF-β1 as same as PA. Furthermore, statistically different IL-10 levels were observed in each group (*P* < 0.05) relative to the model group. On the other hand, significant differences in IL-6 levels between model group and the PA and high-dose FDPGLM groups were detected (*P* < 0.05, *P* < 0.01, respectively), whereas no significant difference between the model group and other groups (*P* > 0.05). These findings suggest that high-dose FDPGLM could significantly reduce the serum levels of the pro-inflammatory cytokine IL-6 in the rat model. The serum IL-17 levels of the model group were significantly different from those of the high-dose group (*P* < 0.01), PA group (*P* < 0.05), and middle-dose group (*P* < 0.05), whereas no statistical significance was observed with low-dose group (*P* > 0.05).

### Differentiation of CD4^+^CD25^+^Foxp3^+^Tregs

**Fig. 3:**
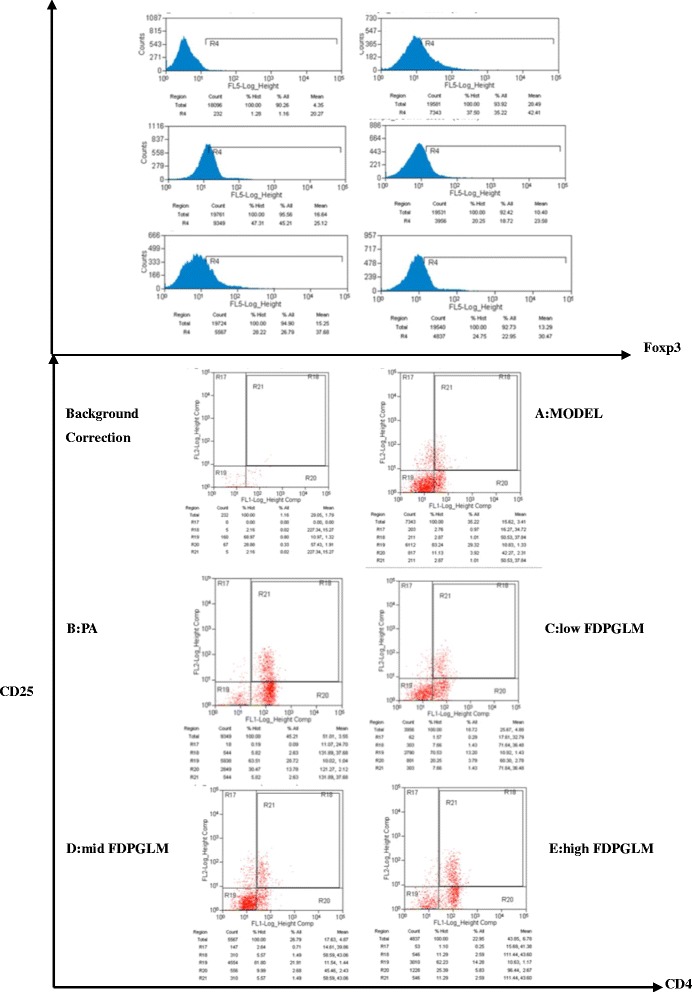
The effect of FDPGLM on the differentiation of CD4^+^CD25^+^Foxp3^+^Tregs in peripheral blood as detected by flow cytometry using three different; fluorescent labels. Representative dot plots from the FC analysis of three staining for CD4, CD25 and foxp3. Lymphocytes from peripheral blood were gated based on SSC

**Fig. 4:**
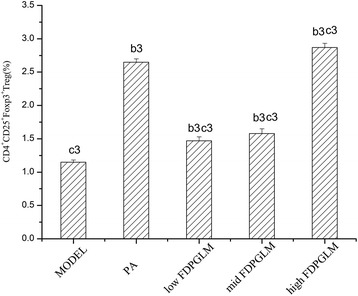
Effect of FDPGLM on the differentiation of CD4^+^CD25^+^Foxp3^+^Tregs in peripheral blood

After 20 days of administration, the CD4^+^CD25^+^Foxp3^+^Tregs/CD4^+^T ratio of ROU rats was assessed (Figs. 3 and 4). ANOVA showed significant differences between the model group (1.15 ± 0.03%) and the other groups (*P* < 0.01). The ratio of CD4^+^CD25^+^Foxp3^+^Tregs to CD4^+^T cells in the PA, low-dose FDPGLM, middle-dose FDPGLM, and high-dose FDPGLM groups were 2.65 ± 0.05%, 1.47 ± 0.06%, 1.58 ± 0.07%, and 2.87 ± 0.06%, respectively. The ratio increased by 1.5-fold in the high-dose FDPGLM group compared to that in the model group. On the other hand, the ratio of the PA group was significantly lower than that in high-dose FDPGLM group (*P* < 0.01), thereby suggesting that high-dose FDPGLM has a stronger effect on enhancing Treg differentiation than that observed using PA.

### Expression of Foxp3 and RORγt transcription factor gene

**Fig. 5:**
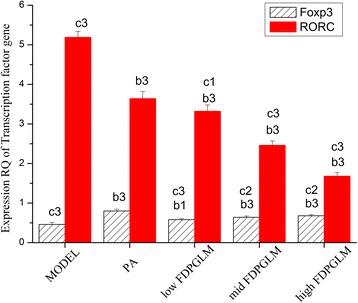
Effect of FDPGLM on the expression of the Foxp3 and RORγt transcription factor gene in pathological tissue

The effect of FDPGLM on the expression of the Foxp3 and RORγt transcription factor gene in ROU rats are shown in Fig. 5. We observed the following: (1): Foxp3: The level of expression of the Foxp3 gene in the PA and middle- and high-dose FDPGLM groups significantly increased (*P* < 0.01) in contrast to that in the model group. In addition, compared to the PA group, the Foxp3 gene expression levels of the middle- and high-dose FDPGLM groups were significantly different (*P* < 0.05). (2) RORγt: There were markedly significant differences between that of the model and the other groups (*P* < 0.01). Therefore, different dosages of FDPGLM can significantly downregulate the expression of the RORγt gene in tissues. Furthermore, statistically significant differences between the PA and the middle- and high-dose groups were observed (*P* < 0.01), indicating that the effects of middle-\and high-dose FDPGLM on RORγt gene expression in tissues are stronger than PA.

### Correlation between CD4^+^CD25^+^Foxp3^+^Tregs and expression of Foxp3 transcription factor gene in high-dose FDPGLM group

**Fig. 6:**
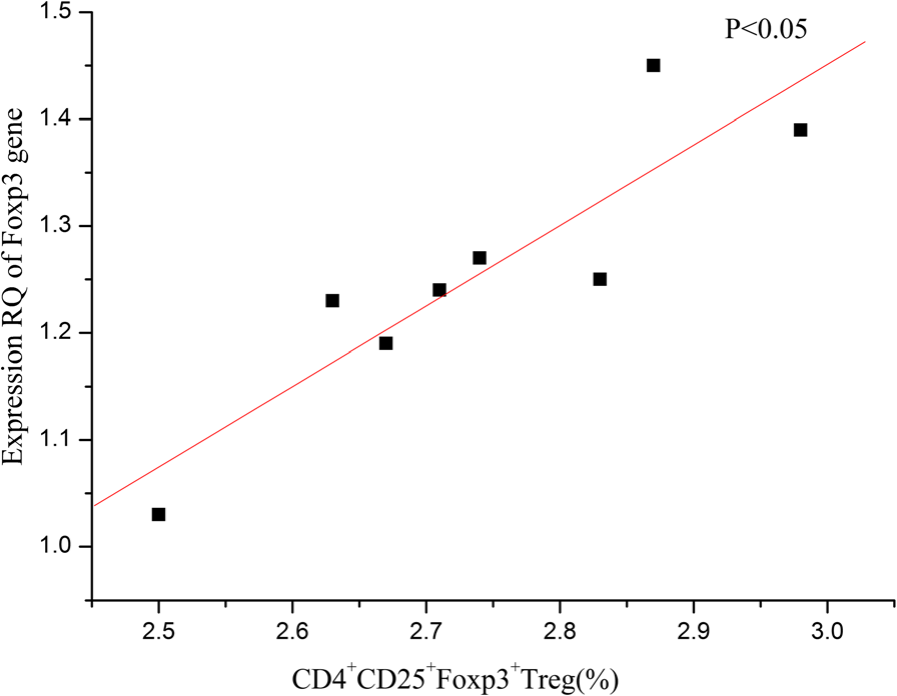
Correlation of Tregs/T ratio and expression of Foxp3 transcription factor gene in high-dose FDPGLM group. The index of Tregs/T ratio was correlated to expression RQ of Foxp3 gene (*P* < 0.05)

We analysed the correlation of the ratio of Treg to T cells to the expression of Foxp3 transcription factor gene in high-dose FDPGLM group.. As shown in Fig. 6, there is a strong inverse correlation between Tregs/T ratio and the expression of Foxp3 transcription factor gene. This result suggests that the increased ratio of Treg to T cells likely reflects the upregulating the expressionof Foxp3 transcription factor gene in high-dose FDPGLM group.

## Discussion

Numerous research studies involving ROU animal models have been conducted around the world; however, one major limitation of most animal models of ROU is that they do not reproduce the chronic or relapsing-remitting pattern characteristic of ROU that is observed in humans. The establishment of an ROU animal model is generally performed by introducing trauma such as the acetic acid method [20] or the No. 15 scalpel blade method [21]. Because these models generally exhibit no relapsing-remitting characteristics and are described as acute inflammations, a very close analogy has been established between the above ROU animal models and traumatic ulcer animal models. With features of chronic, relapsing, and segmental mitigation, ROU behaves more closely to ADs in some instances. Although scientists have not been able to establish a generally accepted and good reproducible animal model for ADs to date, animal models established by using the immune-induced method more closely represented the clinical state [22, 23]. A gamma ray method for induced ROU in an animal model has also been reported [24]. Kaufmann et al. used retinal S- antigen and interphotoreceptor retinoid-binding protein to induce monophasic or relapsing-remitting autoimmune uveitis (EAU), which enabled us to compare autoreactive and regulatory T cell populations [25]. In this study, although the ROU model using SD rats was established by autoantigen injection and exhibited a relapsing-remitting pattern, it also presented the following problems: the time for establishment of the model was too long, and the mortality rate was high. Therefore, additional studies on how to develop a better ROU or AD animal model are warranted.

In our study, the total interval, number of ulcers, and area were investigated in a ROU rat model. Based on these findings, we inferred that high-dose FDPGLM could significantly prolong interval duration and reduce the number of ulcers in ROU, as evaluated according to GSA standards (marked I1N1 in Table 1). The size of the area of the ulcers was also compared to the reference value for therapeutic evaluation, which indicated that the application of high-dose FDPGLM induced a significant reduction (*P* < 0.01).

In our morphological analysis, epithelial thickening in high-dose FDPGLM group was more than model group,with the cells of the basement layer and the lamina propria exhibited normal organization, thereby indicating that high-dose FDPGLM had contributed to the repair of the mucosal membrane and inhibited the infiltration of inflammatory cells.

**Fig. 7:**
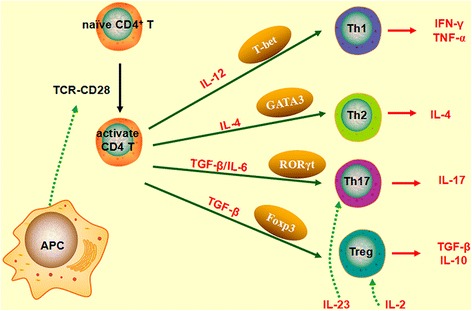
Differentiation of naïve CD4 T cells differentiate into Th (including Th 17) and Treg subsets (designed by using ScienceSlides)

We have mianly examined the effects of FDPGLM on Th17 cells and Tregs in ROU rats.Our understanding of the pathogenesis of ROU has not been fully elucidated, although it is believed to be mediated by an immune response involving T cells. Upon antigen stimulation, naïve CD4^+^ cells differentiated into diverse subsets based on the pattern of cytokines present in the specific environment such as Tregs and Th17 cells [26]. These effector T cells and their interactions determine the direction of immune response in ADs (including ROU).Th17 cells are important mediators of autoimmunity; however, the mechanisms by which these are controlled are not fully understood. IL-6 and TGF-β1 are essential to the differentiation from naïve CD4^+^ T cells into Th17 cells in rats (Fig. 7) [23]. RORγt as a key transcription factor involved in the generation of Th17 cells, which mediate tissue inflammation and autoimmunity [27]. Th17 cells are a pro-inflammatory subset that has been implicated in various inflammatory conditions in humans and rats by producing IL-17 and IL-6. Tregs that co-express CD4, CD25, and Foxp3 are another lineage of CD4^+^ T cells that play a major role in controlling self-reactive T cells to maintain immunologic self-tolerance via contact-dependent suppression [28], and also have an anti-inflammatory by releasing anti-inflammatory cytokines, including IL-10 and TGF-β1 (Fig. 7). The depletion or functional changes in these cytokines also lead to the development of autoimmune disease in animals. Previous studies have shown that the inhibition of CD4^+^CD25^+^Foxp3^+^Tregs result in the functional impairment or decrease in the number of cells in various ADs such as multiple sclerosis (MS) and rheumatoid arthritis (RA) [29, 30].

We investigated the levels of cytokines that are associated with Th17 cells and Tregs in ROU rats(TGF-β1, IL-17, IL-10, and IL-6).Analysis of cytokine production showed that high-dose FDPGLM induced both TGF-β1 and IL-10 production.A report had shown that maintaining a high TGF-β1 serum level enhances differentiation of CD4^+^CD25^+^Foxp3^+^Tregs as well as serves as the trigger for the regulation of immune responses [31]. Our investigation into the cytokine microenvironment of rats (high-dose FDPGLM) also revealed a decrease in IL-17 and IL-6.These findings indicate that the treatment of ROU rats with high-dose FDPGLM improves the levels of the Tregs-associated cytokines (TGF-β1 and IL-10) and decreases those of Th17-associated cells, namely, IL-17 and IL-6.

We also assessed the CD4^+^CD25^+^Foxp3^+^Tregs/CD4^+^T ratio of ROU rats. TGF-β1 was involved in Treg differentiation.At high level of TGF-β1, Treg differentiation is induced, whereas at low level ofTGF-β1, plus IL-6, the differentiation of Th17 cells is promoted [32]. Indeed our findings support this fact.The application of a high-dose FDPGLM resulted in an enhancement in the differentiation of CD4^+^CD25^+^Foxp3^+^Treg cells, which play a major role in immune regulation by inhibiting cellular immunity and the release of inflammatory cytokines.

In RT-PCR analysis, we observed Foxp3 gene expression levels of high-dose FDPGLM groups were significantly different from model groups, indicating that high-dose FDPGLM can significantly upregulate the expression of the Foxp3 gene. Meanwhile, different dosages of FDPGLM can significantly downregulate the expression of the RORγt gene in tissues,especially high-dose FDPGLM. Therefore, high doses of FDPGLM may upregulate the expression of the Foxp3 gene and downregulating the expression of the RORγt gene in ROU rats.

What is the effective substance in FDPGLM? A report showed that a number of fungi, including *G. lucidum* produces β-[1, 3]-glucans, exhibit immunomodulatory properties. Although there is no conventional scientific study that supports the use of β-glucan as a treatment regimen, anectodal evidence suggests that it plays a role in the reduction of pain and recurrence of ROU [12]. Water-soluble polysaccharides (including β-glucans), which are the main bioactive ingredients of *G. lucidum*, are heat sensitive. In the present study, drying of *G. lucidum* mycelia by freeze-drying has retained 2.3-fold more water-soluble polysaccharides compared to using conventional drying.

## Conclusions

The present study investigated the effect of different dosages of FDPGLM on a rat ROU model that was exposed to PA. The results showed that high-dose FDPGLM could significantly prolong total intervals, as well as reduce the number and reduce area of ulcers. Further analysis indicated that high-dose FDPGLM significantly increased the serum levels of TGF-β1, which in turn promoted wound healing, upregulated the expression of the Foxp3 gene, and enhanced CD4^+^CD25^+^Foxp3^+^Tregs differentiation, thereby facilitating immune regulation, inhibition of cellular immunity, and release of inflammatory cytokines. It is possible that high-dose FDPGLM suppressed the differentiation of Th17 cells by downregulating IL-17, IL-6, and RORγt in ROU rats. One possible pathway by which high-dose FDPGLM inhibits the progression of ROU in the rat model is via inhibition of Th17 cell differentiation and inducing Treg differentiation and migration. The differentiation of CD4 T cells into Tregs may contribute in inhibiting effector cells, suppressing immune responses, and improving inflammatory environment in vivo. Furthermore, the observed high serum levels of TGF-β1 might have also contributed to ulcer healing.

## Additional files

Additional file 1: Figure S1. FDPGLM homogenate. Before intragastric administration, FDPGLM dissolved in water and then ground by tissue grinder into homogenate. (DOCX 111 kb)

Additional file 2: Table S1. Content determination of total polysaccharides by UV-Vis spectrophotometry and reproducibility test (*n* = 3). The detection wavelength was 625 nm.Mean content of total polysaccharides was 8.40%(RSD < 5%) and 833.3% higher than the standard in Chinese Pharmacopoeia(≧ 0.9%).RSD:Relative standard deviation. (DOCX 12 kb)

Additional file 3: Table S2. Content determination of triterpenes by UV-Vis spectrophotometry and reproducibility test (*n* = 3). The detection wavelength was 546 nm.Mean content of triterpenes was 0.23%(RSD < 5%) and lower than the standard of CP(≧0.5%). RSD:Relative standard deviation. (DOCX 12 kb)

Additional file 4: Table S3. Content determination of Ganoderic Acid A by HPLC spectrophotometry and reproducibility test (*n* = 3). The content of ganoderic acid A was analysed by HPLC according to the method in the American Herbal Pharmacopoeia and Therapeutic Compendium (Edition 2011). The HPLC conditions were as follows: A chromatographic column of Promosil C18 (4.6 mm × 250 mm, 5 μm) was used, with the mobile phase consisting of 0.1% phosphoric acid-acetonitrile by gradient elution (0–15 min, 20–42%, 15–30 min, 42–60%; 30–35 min, 60%), 1 mL/min flow rate, and 254 nm detection wavelength. HPLC results showed that the content of ganoderic acid A was 1.04‰ (RSD < 5%). RSD:Relative standard deviation. (DOCX 12 kb)

## Abbreviations

ELISA: Enzyme linked immunosorbent assay
FCM: Flow cytometry
FDPGLM: Freeze-dried powder of Ganoderma Lucidum mycelium
Foxp3: Forkhead box P3
IL-17: Interleukin-17
IL-6: Interleukin-6
PA: Prednisone acetate
RORγt: Retinoid-related orphan receptor gamma t
ROU: Recurrent oral ulceration
SD: Sprague-Dawley
TGF-β1: Transforming growth factor-β1
Th 17 cells: T helper cells 17
Tregs: Regulatory T cells
