# Supplementary figures and images for: The effects of freeze-dried Ganoderma lucidum mycelia on a recurrent oral ulceration rat model
Source: BMC Complement Altern Med. 2017 Dec 1;17:511. doi: 10.1186/s12906-017-2021-8 (PMC5709989; doi:10.1186/s12906-017-2021-8)

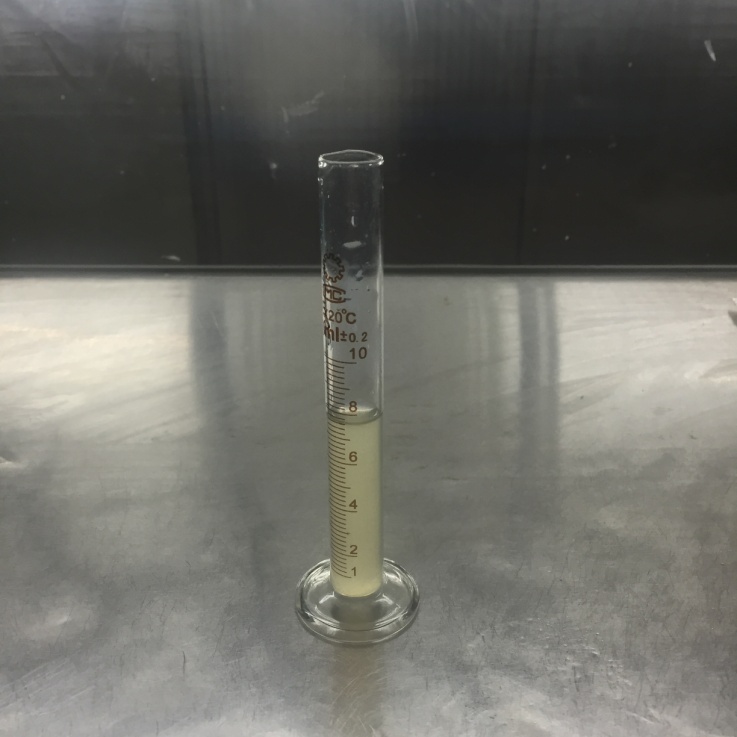


**Supplementary Fig.1**

Supplement: Supplementary file 1 — FDPGLM homogenate. Before intragastric administration, FDPGLM dissolved in water and then ground by tissue grinder into homogenate. (DOCX 111 kb) [file 12906_2017_2021_MOESM1_ESM.docx]
