# Supplementary material for: The effects of freeze-dried Ganoderma lucidum mycelia on a recurrent oral ulceration rat model
Source: BMC Complement Altern Med. 2017 Dec 1;17:511. doi: 10.1186/s12906-017-2021-8 (PMC5709989; doi:10.1186/s12906-017-2021-8)
Supplement: Supplementary file 2 — Content determination of total polysaccharides by UV-Vis spectrophotometry and reproducibility test (n = 3). The detection wavelength was 625 nm.Mean content of total polysaccharides was 8.40%(RSD < 5%) and 833.3% higher than the standard in Chinese Pharmacopoeia(≧ 0.9%).RSD:Relative standard deviation. (DOCX 12 kb) [file 12906_2017_2021_MOESM2_ESM.docx]

**Supplementary Table 1**

| **Sample amount(g)** | **Total Polysaccharides(g)** |  | **Content(%)** | **RSD(%)** | |
| --- | --- | --- | --- | --- | --- |
| 1.0005 | 0.0862 | | 8.61 | 1.93 |  |
| 1.0001 | 0.0854 | | 8.54 | 3.61 |  |
| 1.0006 | 0.0834 | | 8.33 | 1.30 |  |
| 1.0001 | 0.0821 | | 8.21 | 2.06 |  |
| 1.0006 | 0.0834 | | 8.33 | 2.62 |  |
