# Supplementary material for: The effects of freeze-dried Ganoderma lucidum mycelia on a recurrent oral ulceration rat model
Source: BMC Complement Altern Med. 2017 Dec 1;17:511. doi: 10.1186/s12906-017-2021-8 (PMC5709989; doi:10.1186/s12906-017-2021-8)
Supplement: Supplementary file 3 — Content determination of triterpenes by UV-Vis spectrophotometry and reproducibility test (n = 3). The detection wavelength was 546 nm.Mean content of triterpenes was 0.23%(RSD < 5%) and lower than the standard of CP(≧0.5%). RSD:Relative standard deviation. (DOCX 12 kb) [file 12906_2017_2021_MOESM3_ESM.docx]

**Supplementary Table 2**

| **Sample amount(g)** | **Triterpenes(mg)** |  | **Content(%)** | **RSD(%)** | |
| --- | --- | --- | --- | --- | --- |
| 1.0002 | 2.3786 | | 0.24 | 3.95 |  |
| 1.0003 | 2.4009 | | 0.24 | 4.02 |  |
| 1.0003 | 2.5435 | | 0.25 | 3.67 |  |
| 1.0001 | 2.1389 | | 0.21 | 3.03 |  |
| 1.0006 | 2.0712 | | 0.21 | 3.62 |  |
