# Supplementary material for: The effects of freeze-dried Ganoderma lucidum mycelia on a recurrent oral ulceration rat model
Source: BMC Complement Altern Med. 2017 Dec 1;17:511. doi: 10.1186/s12906-017-2021-8 (PMC5709989; doi:10.1186/s12906-017-2021-8)
Supplement: Supplementary file 4 — Content determination of Ganoderic Acid A by HPLC spectrophotometry and reproducibility test (n = 3). The content of ganoderic acid A was analysed by HPLC according to the method in the American Herbal Pharmacopoeia and Therapeutic Compendium (Edition 2011). The HPLC conditions were as follows: A chromatographic column of Promosil C18 (4.6 mm × 250 mm, 5 μm) was used, with the mobile phase consisting of 0.1% phosphoric acid-acetonitrile by gradient elution (0–15 min, 20–42%, 15–30 min, 42–60%; 30–35 min, 60%), 1 mL/min flow rate, and 254 nm detection wavelength. HPLC results showed that the content of ganoderic acid A was 1.04‰ (RSD < 5%). RSD:Relative standard deviation. (DOCX 12 kb) [file 12906_2017_2021_MOESM4_ESM.docx]

**Supplementary Table 3**

| **Sample amount(g)** | **Ganoderic Acid A(mg)** |  | **Content(‰)** | **RSD(%)** | |
| --- | --- | --- | --- | --- | --- |
| 1.0003 | 1.0762 | | 1.08 | 4.78 |  |
| 1.0000 | 0.9869 | | 0.99 | 4.42 |  |
| 1.0004 | 1.0952 | | 1.09 | 3.75 |  |
| 1.0002 | 0.9945 | | 0.99 | 3.26 |  |
| 1.0005 | 1.0521 | | 1.05 | 4.50 |  |
